# Supplementary material for: IL-27 limits HSPC differentiation during infection and protects from stem cell exhaustion
Source: bioRxiv. 2025 Jun 24:2025.01.15.633135. Originally published 2025 Jan 19. Preprint. [Version 2] doi: 10.1101/2025.01.15.633135 (PMC11761129; doi:10.1101/2025.01.15.633135)

bioRxiv preprint doi: https://doi.org/10.1101/2025.01.15.633135; this version posted June 24, 2025. The copyright holder for this preprint (which was not certified by peer review) is the author/funder, who has granted bioRxiv a license to display the preprint in perpetuity. It is made available under aCC-BY 4.0 International license.

Supplemental Figure 1

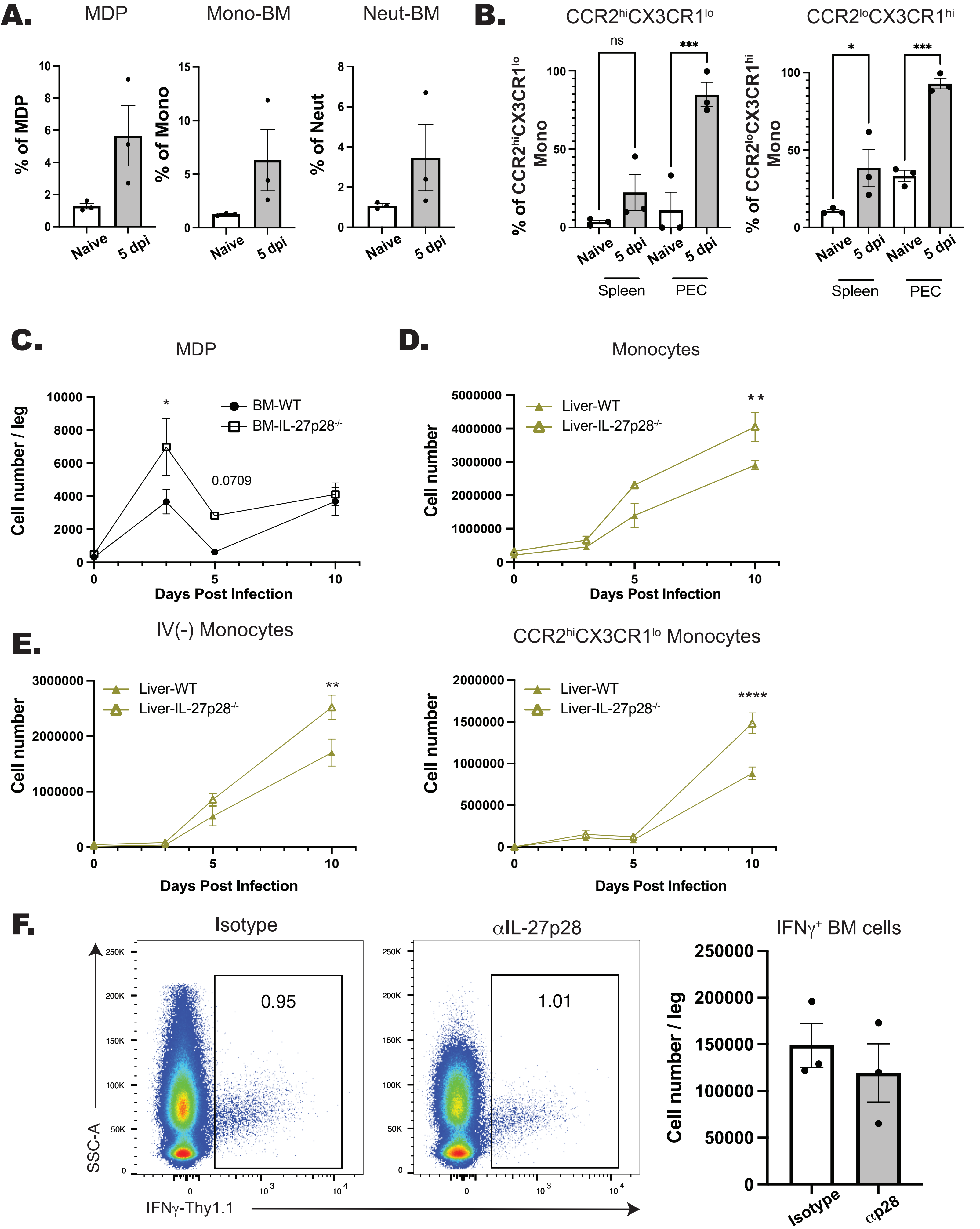

bioRxiv preprint doi: https://doi.org/10.1101/2025.01.15.633135; this version posted June 24, 2025. The copyright holder for this preprint (which was not certified by peer review) is the author/funder, who has granted bioRxiv a license to display the preprint in perpetuity. It is made available under aCC-BY 4.0 International license.

Supplemental Figure 2

Bone marrow gating strategy

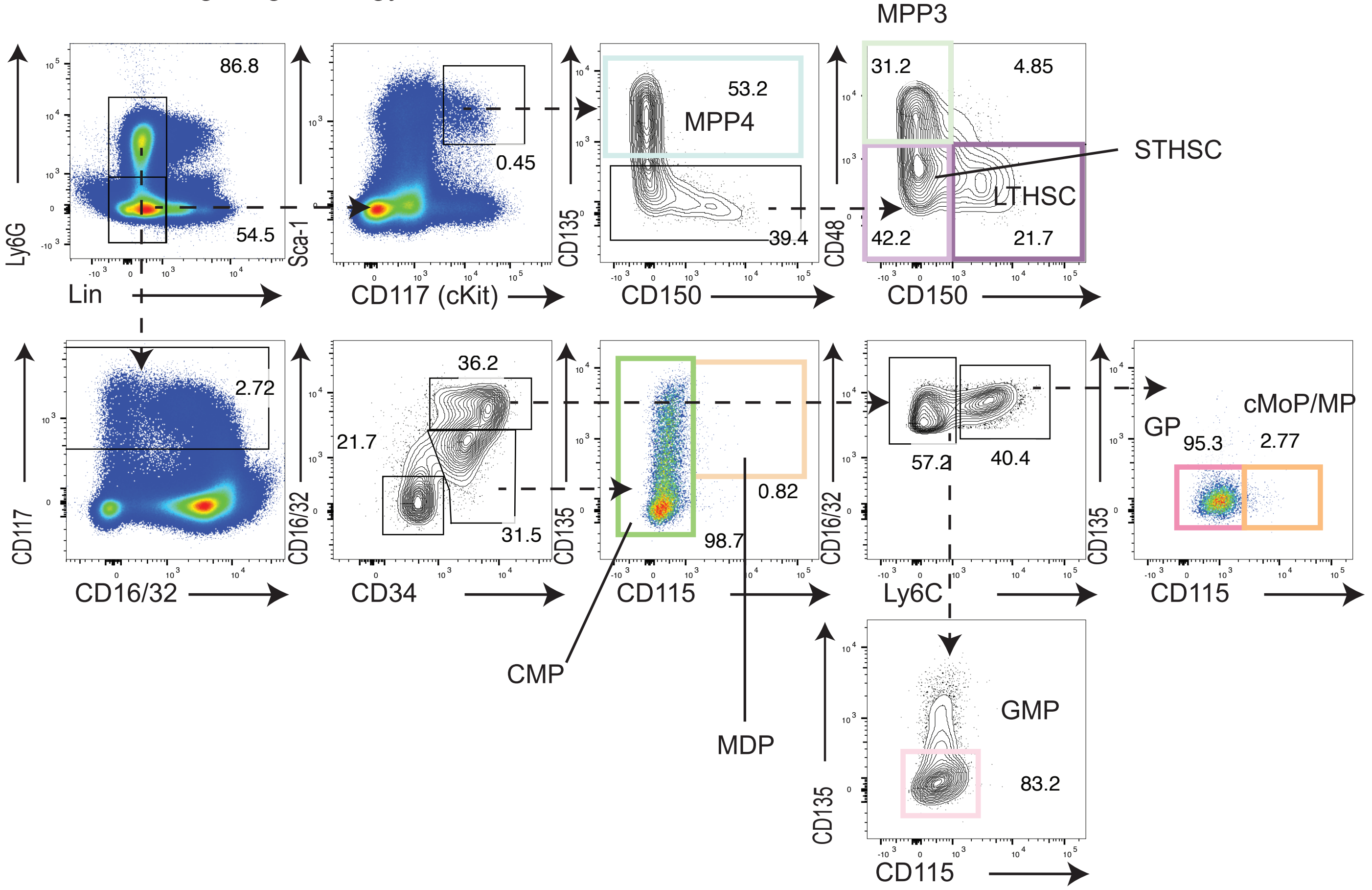

Periphery gating strategy

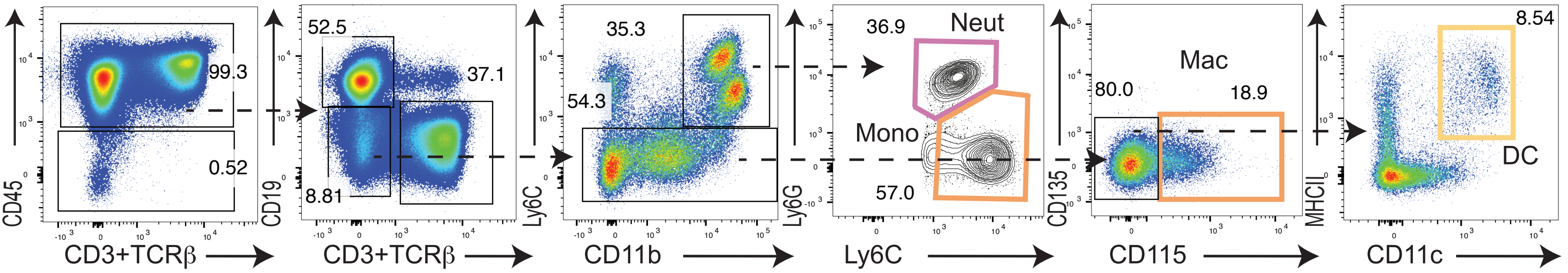

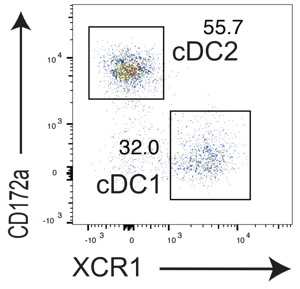

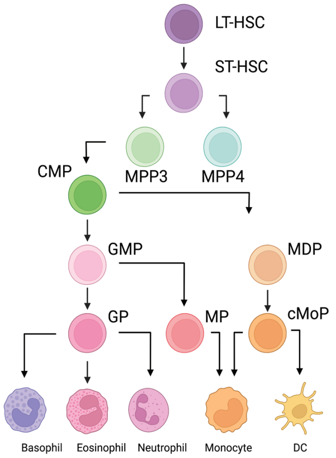

Bone marrow surface markers

Cell Type	Defining Surface Markers
Long-Term Hematopoietic Stem Cell (LTHSC)	Lin (CD3, B220, NK1.1, Ly6G)$^{-}$, Sca-1^{+}, CD117^{+}, CD135^{-}, CD48^{-}, CD150^{+}
Short-Term Hematopoietic Stem Cell (STHSC)	Lin (CD3, B220, NK1.1, Ly6G)$^{-}$, Sca-1^{+}, CD117^{+}, CD135^{-}, CD48^{-}, CD150^{-}
Multi-Potent Progenitor 3 (MPP3)	Lin (CD3, B220, NK1.1, Ly6G)$^{-}$, Sca-1^{+}, CD117^{+}, CD135^{-}, CD48^{+}, CD150^{-}
Multi-Potent Progenitor 4 (MPP4)	Lin (CD3, B220, NK1.1, Ly6G)$^{-}$, Sca-1^{+}, CD117^{+}, CD135^{+}
Common Myeloid Progenitor (CMP)	(CD3, B220, NK1.1)$^{-}$, CD117^{+}, CD34^{+}, CD16/32^{lo}, CD115^{-}
Monocyte-Dendritic cell Progenitor (MDP)	(CD3, B220, NK1.1)$^{-}$, CD117^{+}, CD34^{+}, CD16/32^{lo}, CD115^{+}
Common Monocyte Progenitor (cMoP)/ Monocyte Progenitor (MP)	(CD3, B220, NK1.1)$^{-}$, CD117^{+}, CD34^{+}, CD16/32^{hi}, Ly6C^{+}, CD135^{-}, CD115^{+}
Granulocyte-Monocyte Progenitor (GMP)	(CD3, B220, NK1.1)$^{-}$, CD117^{+}, CD34^{+}, CD16/32^{hi}, Ly6C^{-}, CD135^{-}, CD115^{-}
Granulocyte Progenitor (GP)	(CD3, B220, NK1.1)$^{-}$, CD117^{+}, CD34^{+}, CD16/32^{hi}, Ly6C^{+}, CD135^{-}, CD115^{-}

Periphery surface markers

Cell Type	Defining Surface Markers
T cells	CD45^{+}, CD19^{-}, CD3^{+}, TCRβ^{+}
B cells	CD45^{+}, CD19^{+}, CD3^{-}, TCRβ^{-}
Neutrophils	CD45^{+}, CD19^{-}, CD3^{-}, TCRβ^{-}, CD11b^{+}, Ly6C^{+}, Ly6G^{+}
Monocytes	CD45^{+}, CD19^{-}, CD3^{-}, TCRβ^{-}, CD11b^{+}, Ly6C^{+}, Ly6G^{-}
Macrophages	CD45^{+}, CD19^{-}, CD3^{-}, TCRβ^{-}, Ly6C^{-}, Ly6G^{-}, CD64^{+}
Dendritic Cells	CD45^{+}, CD19^{-}, CD3^{-}, TCRβ^{-}, Ly6C^{-}, Ly6G^{-}, CD64^{-}, CD11c^{+}, MHCII^{+}
Classical Dendritic Cells 1	CD45^{+}, CD19^{-}, CD3^{-}, TCRβ^{-}, Ly6C^{-}, Ly6G^{-}, CD64^{-}, CD11c^{+}, MHCII^{+}, XCR1^{+}, CD172a^{-}
Classical Dendritic Cells 2	CD45^{+}, CD19^{-}, CD3^{-}, TCRβ^{-}, Ly6C^{-}, Ly6G^{-}, CD64^{-}, CD11c^{+}, MHCII^{+}, XCR1^{+}, CD172a^{-}

bioRxiv preprint doi: https://doi.org/10.1101/2025.01.15.633135; this version posted June 24, 2025. The copyright holder for this preprint (which was not certified by peer review) is the author/funder, who has granted bioRxiv a license to display the preprint in perpetuity. It is made available under aCC-BY 4.0 International license.

Supplemental Figure 3

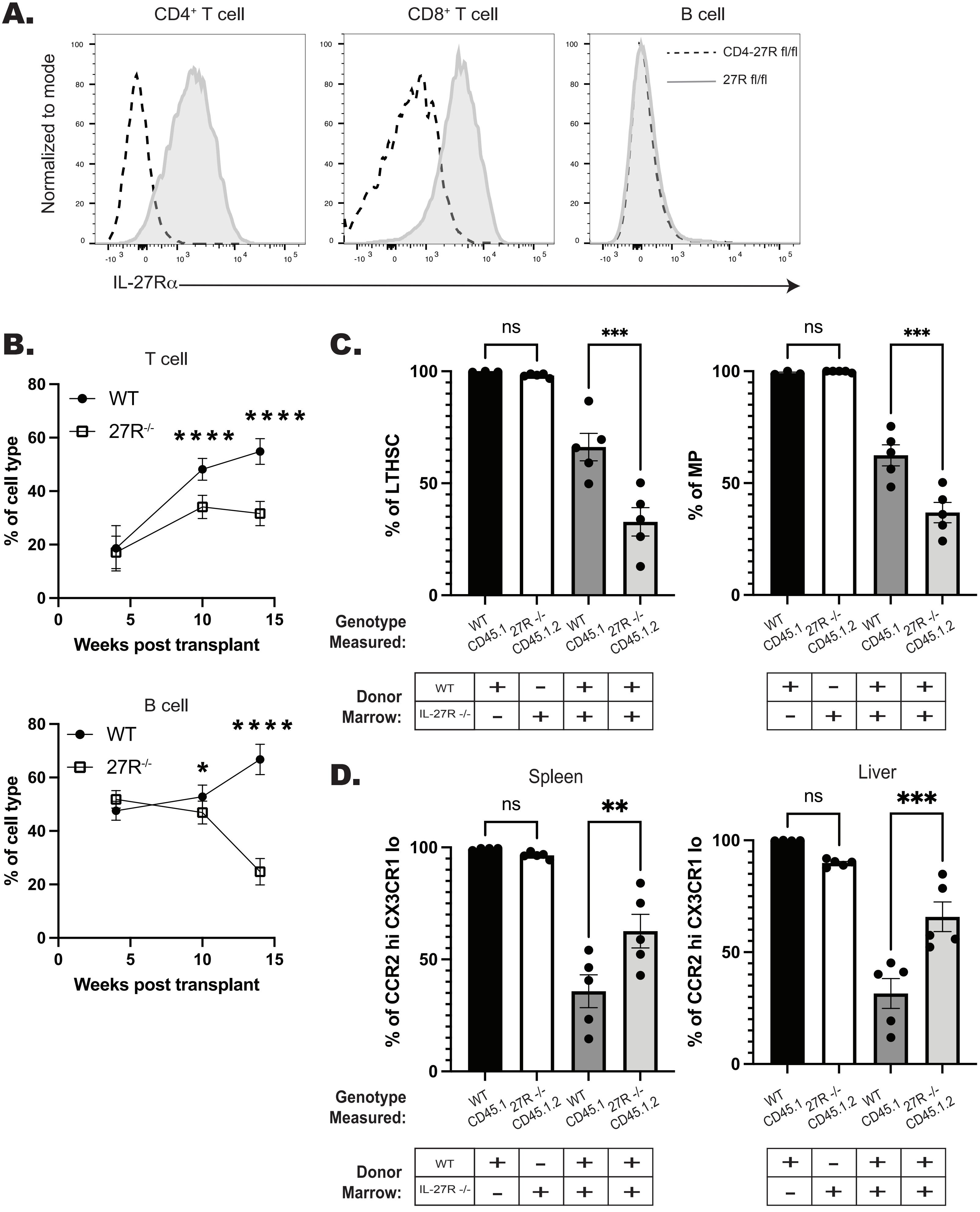

bioRxiv preprint doi: https://doi.org/10.1101/2025.01.15.633135; this version posted June 24, 2025. The copyright holder for this preprint (which was not certified by peer review) is the author/funder, who has granted bioRxiv a license to display the preprint in perpetuity. It is made available under aCC-BY 4.0 International license.

Supplemental Figure 4

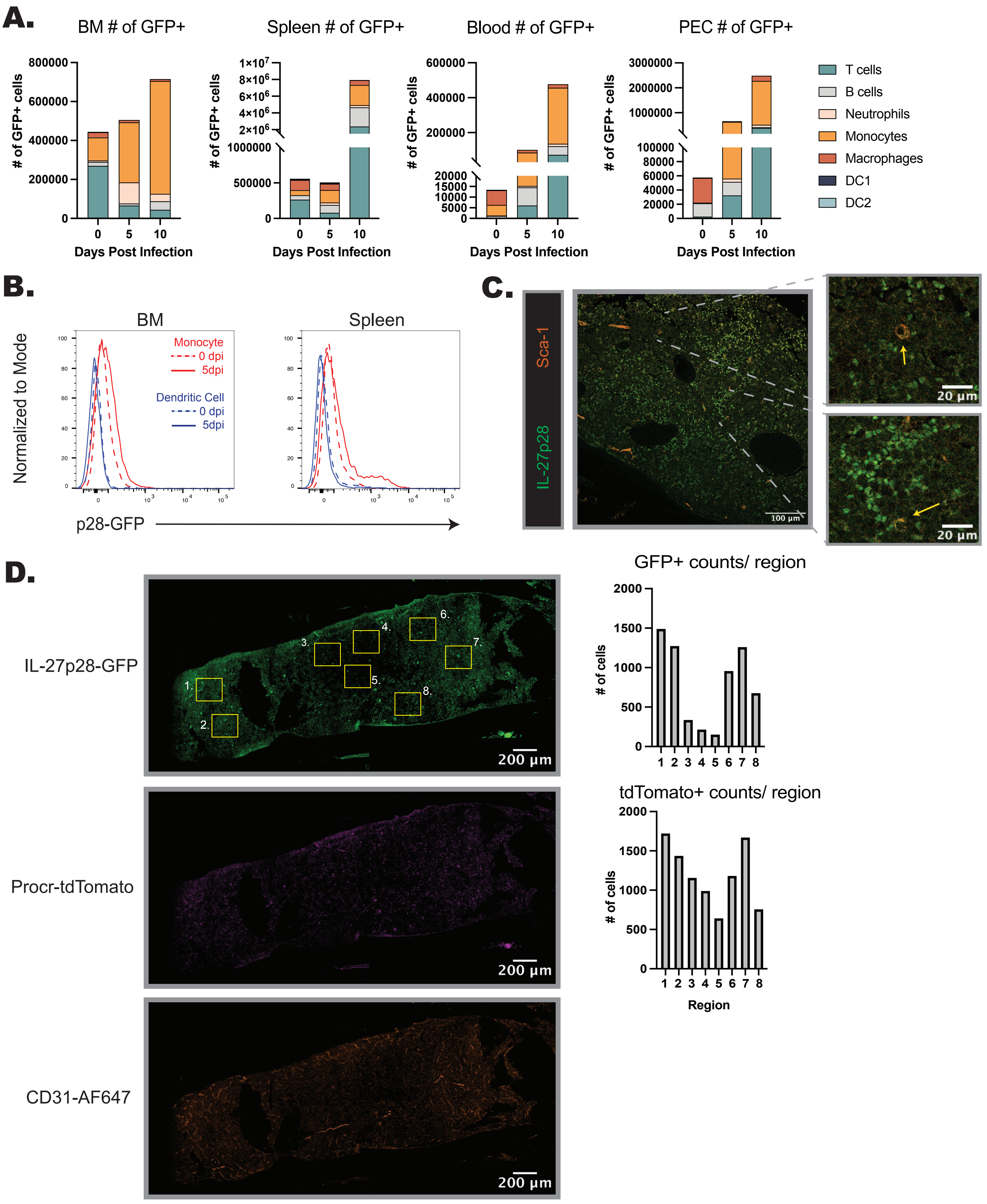

bioRxiv preprint doi: https://doi.org/10.1101/2025.01.15.633135; this version posted June 24, 2025. The copyright holder for this preprint (which was not certified by peer review) is the author/funder, who has granted bioRxiv a license to display the preprint in perpetuity. It is made available under aCC-BY 4.0 International license.

Supplemental Figure 5

A.

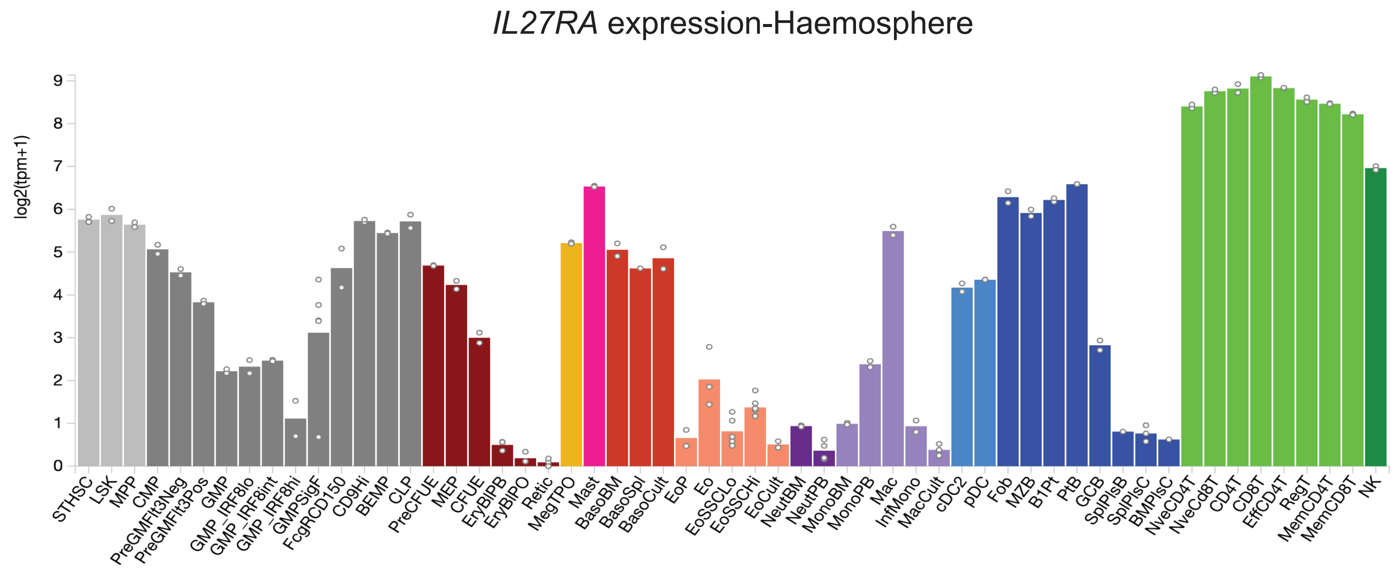

B.

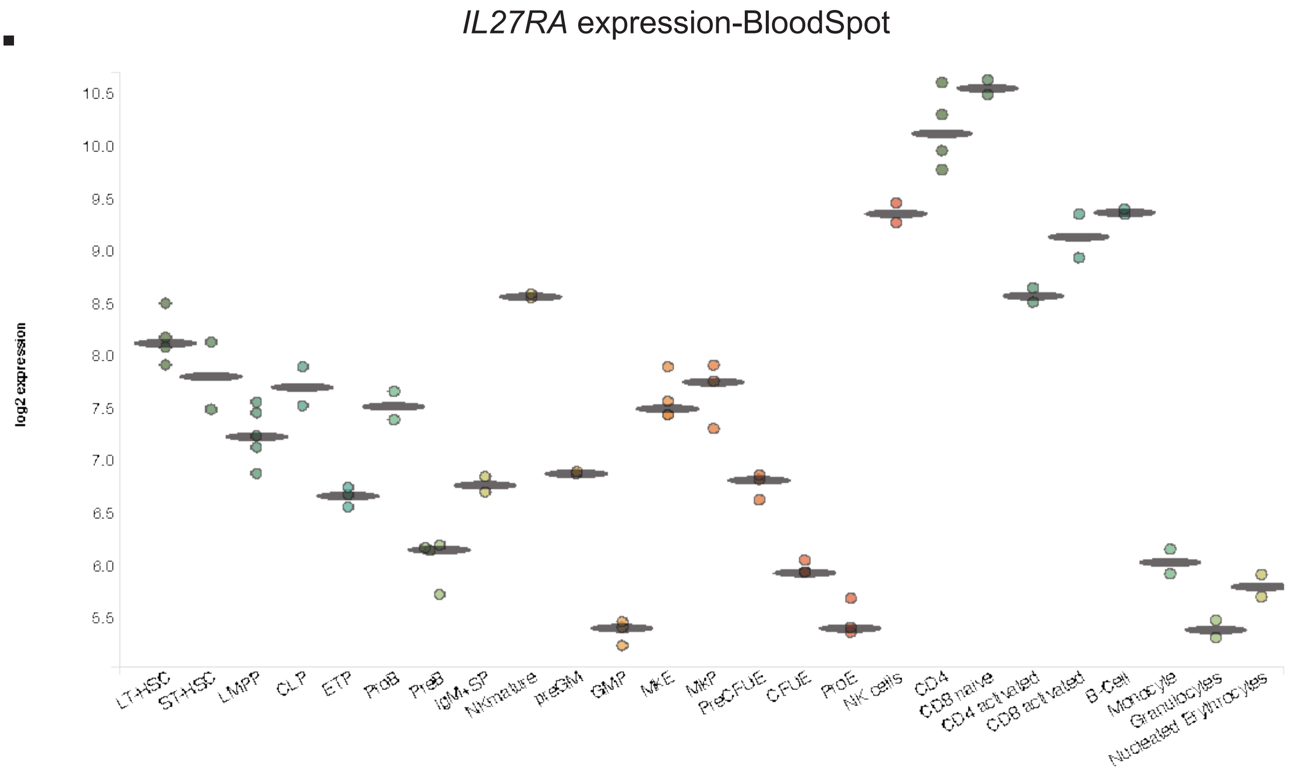

C.

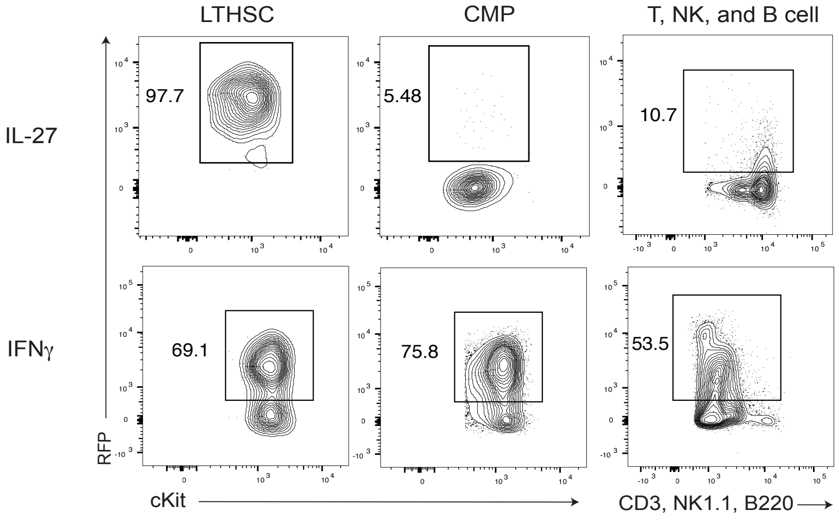

bioRxiv preprint doi: https://doi.org/10.1101/2025.01.15.633135; this version posted June 24, 2025. The copyright holder for this preprint (which was not certified by peer review) is the author/funder, who has granted bioRxiv a license to display the preprint in perpetuity. It is made available under aCC-BY 4.0 International license.

Supplemental Figure 6

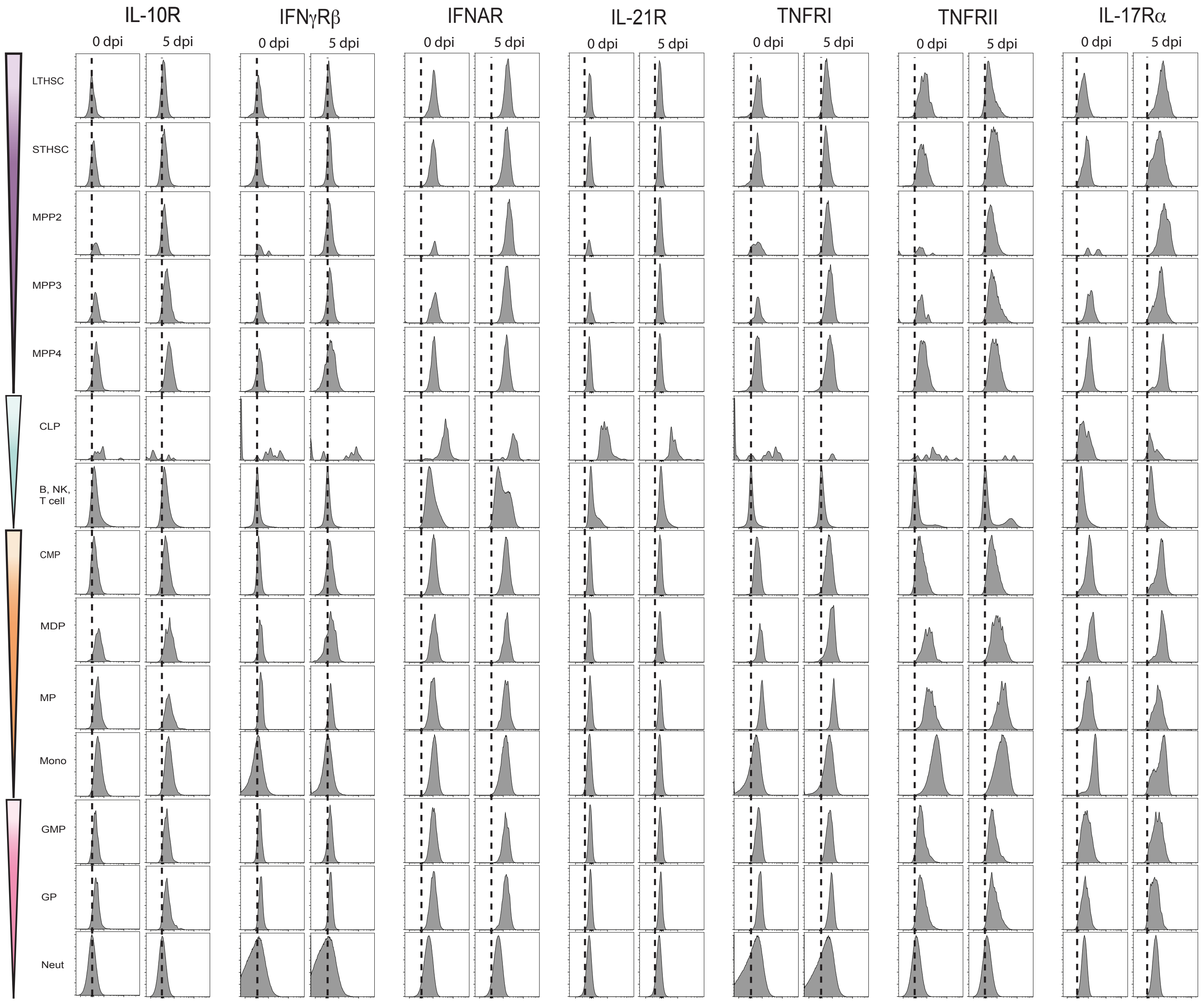

bioRxiv preprint doi: https://doi.org/10.1101/2025.01.15.633135; this version posted June 24, 2025. The copyright holder for this preprint (which was not certified by peer review) is the author/funder, who has granted bioRxiv a license to display the preprint in perpetuity. It is made available under aCC-BY 4.0 International license.

Supplemental Figure 7

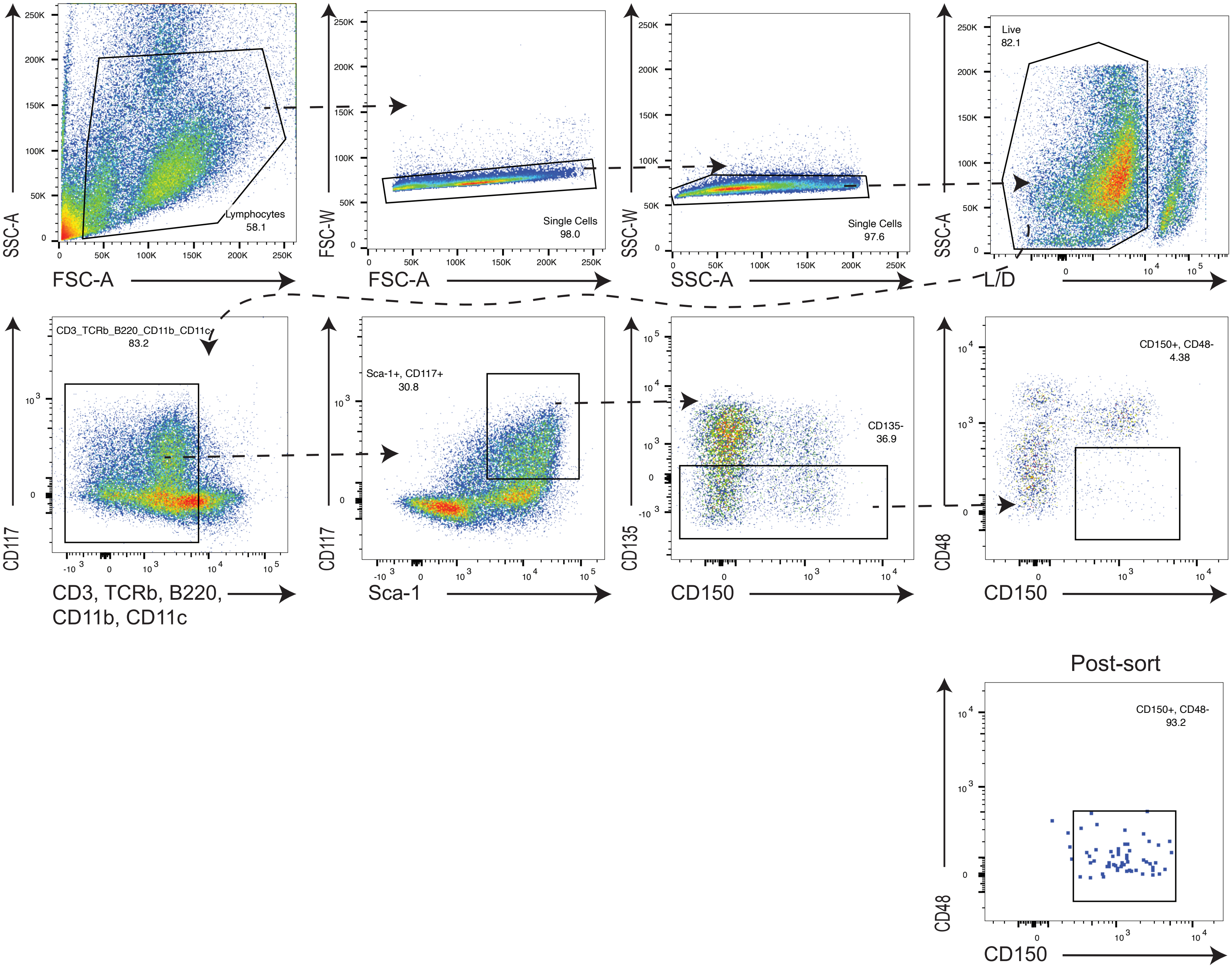

bioRxiv preprint doi: https://doi.org/10.1101/2025.01.15.633135; this version posted June 24, 2025. The copyright holder for this preprint (which was not certified by peer review) is the author/funder, who has granted bioRxiv a license to display the preprint in perpetuity. It is made available under aCC-BY 4.0 International license.

Supplemental Figure 8

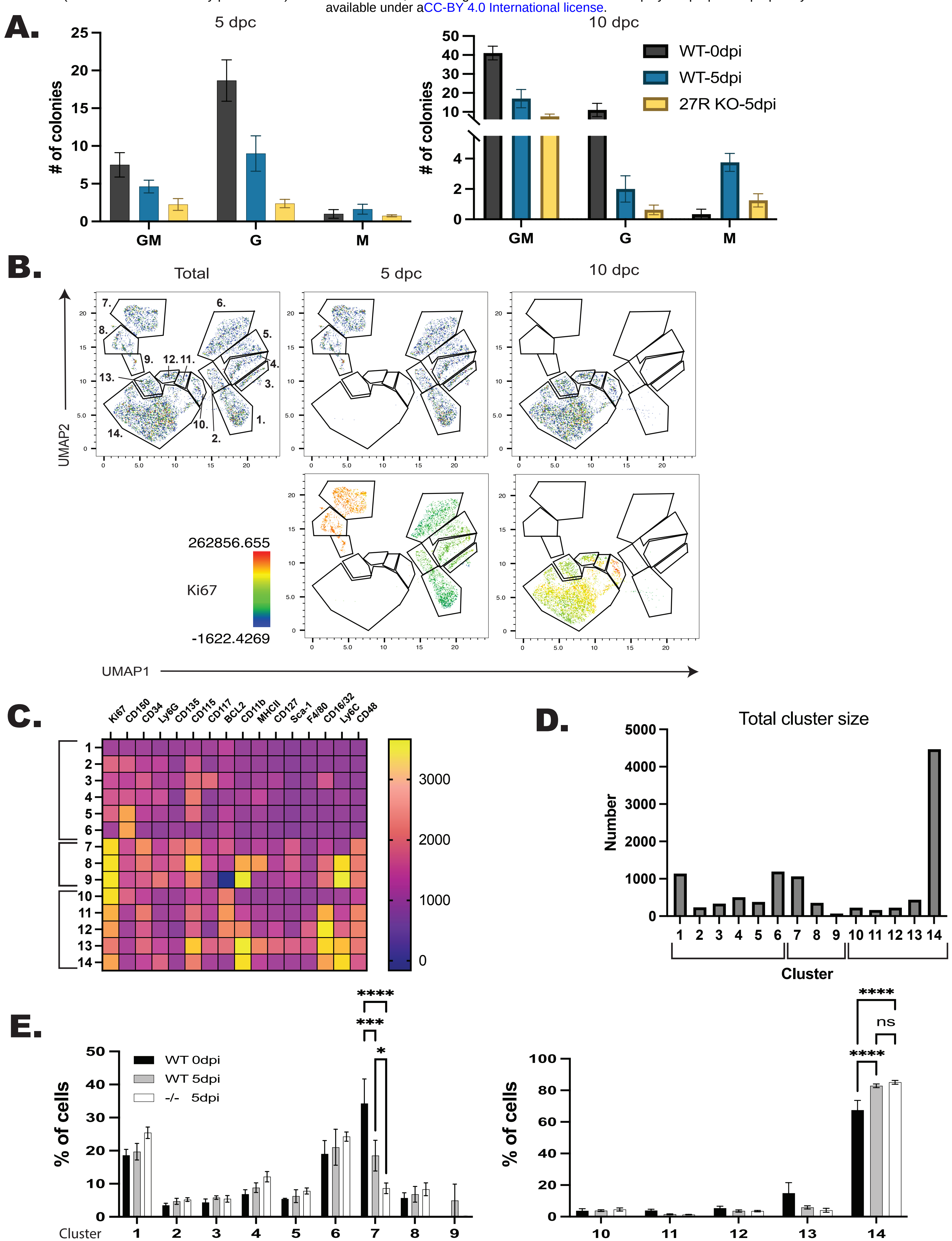

bioRxiv preprint doi: https://doi.org/10.1101/2025.01.15.633135; this version posted June 24, 2025. The copyright holder for this preprint (which was not certified by peer review) is the author/funder, who has granted bioRxiv a license to display the preprint in perpetuity. It is made available under aCC-BY 4.0 International license.

Supplemental Figure 9

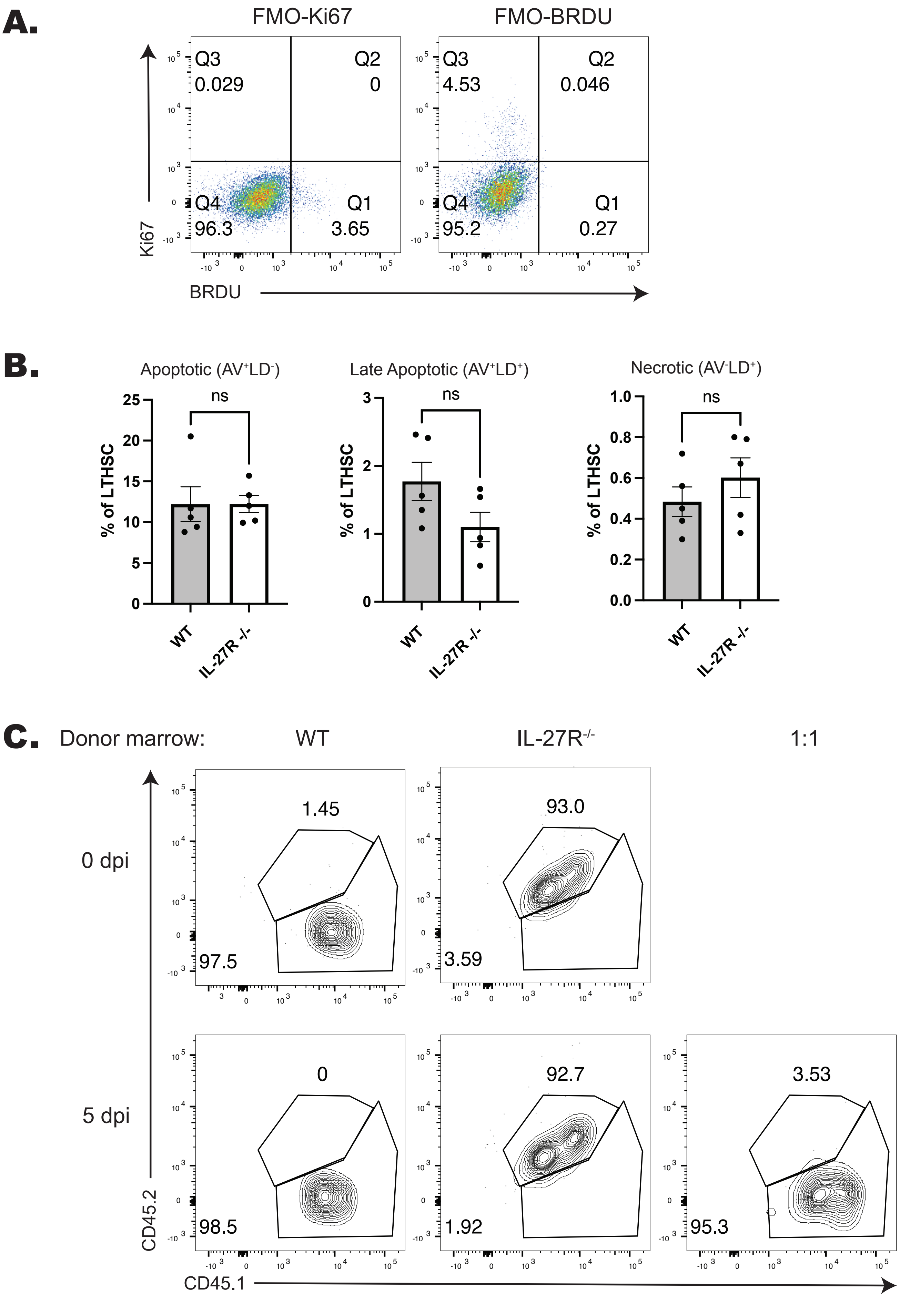

Supplement: 1 — Supplemental Figure 1. IL-27 regulates monopoiesis during infection. A) The proportion of zsGreen+ monocyte dendritic cell progenitors (MDPs) (CD3−, NK1.1−, B220−, CD117+, CD34+, CD16/32lo, CD115+), monocytes, and neutrophils (CD3−, B220−, CD11b+, Ly6C+, Ly6G+) in the BM of naïve and infected Procr-Ai6 mice. B) The proportion of zsGreen+ CCR2hi/lo CX3CR1hi/lo monocytes in the spleen and PECs in naïve and infected Procr-Ai6 mice. C) Number of MDPs in WT and IL-27p28−/− mice in the bone marrow during infection. D) Numbers of monocytes in the liver in WT and IL-27p28−/− throughout infection. E) WT and IL-27p28−/− mice were injected I.V. with fluorescent anti-CD45 to label immune cells in the vasculature. Numbers of I.V. label− and CCR2hiCX3CR1lo monocytes in the liver were then quantified throughout infection. F) IFNγ-Thy1.1 reporter mice were infected and treated with an anti-p28 blocking antibody. Numbers of IFNγ+ cells in the bone marrow was then assessed by flow cytometry. Representative flow plots are shown (left) and quantified (right). Statistical significance was tested by one-way ANOVA with Sidak’s correction. *, **, and *** correspond to p-values ≤ 0.05, 0.01, and 0.001, respectively. N=3–5 mice/group and data shown are representative of 2–3 experiments. Supplemental Figure 2. Gating strategy for flow cytometric analysis of cells in the bone marrow and periphery. Representative flow plots from the bone marrow (top) and spleen (bottom) are shown indicating the gating strategy used to identify various cell populations. The combination of surface markers used are listed in the tables (bottom) and are color coded to correspond to both the developmental schematic pictured as well as the corresponding flow gate on the representative plots. Supplemental Figure 3. IL-27 regulates monopoiesis during infection and post-irradiation in a cell intrinsic manner. A) IL-27Rα expression on CD4+ (CD19−CD3+CD8α−) T cells, CD8+ (CD19−CD3+CD4−) T cells, and B cells (CD19+CD3−) in [file NIHPP2025.01.15.633135V2-supplement-1.pdf]
