# Supplementary material for: IL-27 limits HSPC differentiation during infection and protects from stem cell exhaustion
Source: bioRxiv. 2025 Jan 19:2025.01.15.633135. Preprint. [Version 1] doi: 10.1101/2025.01.15.633135 (PMC11761129; doi:10.1101/2025.01.15.633135)

bioRxiv preprint doi: https://doi.org/10.1101/2025.01.15.633135; this version posted January 19, 2025. The copyright holder for this preprint (which was not certified by peer review) is the author/funder, who has granted bioRxiv a license to display the preprint in perpetuity. It is made available under aCC-BY 4.0 International license.

Figure legends

Supplemental Figure 1. IL-27 regulates monopoiesis during infection. A) The proportion of zsGreen$^+$ monocyte dendritic cell progenitors (MDPs) (CD3$^-$, NK1.1$^-$, B220$^-$, CD117$^+$, CD34$^+$, CD16/32^{lo}, CD115$^+$), monocytes, and neutrophils (CD3$^-$, B220$^-$, CD11b$^+$, Ly6C$^+$, Ly6G$^+$) in the BM of naïve and infected Procr-Ai6 mice. B) The proportion of zsGreen$^+$ CCR2$^{hi/lo}$ CX3CR1$^{hi/lo}$ monocytes in the spleen and PECs in naïve and infected Procr-Ai6 mice. C) Number of MDPs in WT and IL-27p28$^{-/-}$ mice in the bone marrow during infection. D) Numbers of monocytes in the liver in WT and IL-27p28$^{-/-}$ throughout infection. E) WT and IL-27p28$^{-/-}$ mice were injected I.V.

bioRxiv preprint doi: https://doi.org/10.1101/2025.01.15.633135; this version posted January 19, 2025. The copyright holder for this preprint (which was not certified by peer review) is the author/funder, who has granted bioRxiv a license to display the preprint in perpetuity. It is made available under aCC-BY 4.0 International license.

with fluorescent anti-CD45 to label immune cells in the vasculature. Numbers of I.V. label^{-} and CCR2^{hi}CX3CR1^{lo} monocytes in the liver were then quantified throughout infection. Statistical significance was tested by one-way ANOVA with Sidak's correction. *, **, and *** correspond to p-values ≤ 0.05, 0.01, and 0.001, respectively. N=3-5 mice/group and data shown are representative of 2-3 experiments.

Supplemental Figure 2. Gating strategy for flow cytometric analysis of cells in the bone marrow and periphery. Representative flow plots from the bone marrow (top) and spleen (bottom) are shown indicating the gating strategy used to identify various cell populations. The combination of surface markers used are listed in the tables (bottom) and are color coded to correspond to both the developmental schematic pictured as well as the corresponding flow gate on the representative plots.

bioRxiv preprint doi: https://doi.org/10.1101/2025.01.15.633135; this version posted January 19, 2025. The copyright holder for this preprint (which was not certified by peer review) is the author/funder, who has granted bioRxiv a license to display the preprint in perpetuity. It is made available under aCC-BY 4.0 International license.

Supplemental Figure 3. IL-27 regulates monopoiesis during infection and post-irradiation in a cell intrinsic manner. A) IL-27Rα expression on $CD4^{+}$ ($CD19^{-}CD3^{+}CD8\alpha^{-}$) T cells, $CD8^{+}$ ($CD19^{-}CD3^{+}CD4^{-}$) T cells, and B cells ($CD19^{+}CD3^{-}$) in CD4-27R mice at 5 dpi. B) The proportion of LTHSCs (left) and MPs (right) from each respective donor lineage in the bone marrow post-reconstitution and pre-infection. C) The proportion of $CCR2^{hi}CX3CR1^{lo}$ monocytes from each respective donor lineage in the spleen and liver post-reconstitution and pre-infection. Statistical significance was tested by one-way ANOVA with Sidak's correction. *, **, and *** correspond to p-values ≤ 0.05, 0.01, and 0.001, respectively. N=3-5 mice/group and data shown are representative of 2 repeated experiments.

bioRxiv preprint doi: https://doi.org/10.1101/2025.01.15.633135; this version posted January 19, 2025. The copyright holder for this preprint (which was not certified by peer review) is the author/funder, who has granted bioRxiv a license to display the preprint in perpetuity. It is made available under aCC-BY 4.0 International license.

Supplemental Figure 4. IL-27p28 is produced in the bone marrow during infection. A) IL-27p28-GFP mice were infected, and the numbers of each CD45$^+$, immune cell type that expressed GFP in the BM or periphery quantified throughout infection. B) Levels of IL-27p28-GFP expression were measured in bulk monocytes (red) and dendritic cells (blue) in the bone marrow (left) and spleen (right). C) Femurs from naïve IL-27p28-GFP mice were stained for Sca-1 and imaged at 40x magnification. Zoomed in images are shown (right) of the indicated areas, with the yellow arrows indicating positively stained, Sca-1$^+$ cells. D) The merged image shown in Fig. 3D is separated by channel (GFP, tdTomato, and AF647, from top to bottom). Eight randomly selected regions that were used for quantification of either GFP$^+$ or tdTomato$^+$ cells are shown (top), and the quantification of cells in each region is shown next to the corresponding florescence channel (right).

bioRxiv preprint doi: https://doi.org/10.1101/2025.01.15.633135; this version posted January 19, 2025. The copyright holder for this preprint (which was not certified by peer review) is the author/funder, who has granted bioRxiv a license to display the preprint in perpetuity. It is made available under aCC-BY 4.0 International license.

Supplemental Figure 5. IL-27Rα transcript expression during hematopoiesis and differential sensitivity of LTHSC and CMP to IL-27. A) Expression levels of *IL27RA* are shown from the Haemosphere RNA-seq data set and online tool as well as the Blood Spot data set and visualizer (B). C) Representative flow plots of IRGM1-RFP expression in LTHSCs and CMPs 48 hrs. post-stimulation with IL-27.

Supplemental Figure 6. Developmental expression of additional cytokine receptors. Indicated cytokine receptors were analyzed in naive and infected mice as described in Fig. 4-5.

bioRxiv preprint doi: https://doi.org/10.1101/2025.01.15.633135; this version posted January 19, 2025. The copyright holder for this preprint (which was not certified by peer review) is the author/funder, who has granted bioRxiv a license to display the preprint in perpetuity. It is made available under aCC-BY 4.0 International license.

Supplemental Figure 7. Gating strategy for sorting of LTHSCs used in monocyte differentiation. LTHSCs were sorted from WT naïve, WT 5 dpi, and IL-27R$^{-/-}$ 5 dpi mice. The gating strategy is shown as well as the purity of sorted samples (last panel).

Supplemental Figure 8. Development and validation of an unbiased flow cytometry approach for the analysis of MethoCult colonies. WT and IL-27R$^{-/-}$ mice were infected, LTHSCs sorted and cultured in MethoCult for either 5 or 10 days. A) Colony phenotypes were analyzed by microscopy and counted by eye. B) Colonies were collected at either 5 or 10 days post-culture (dpc) and analyzed by flow cytometry. 500 live cells from n=3-4 mice/ group were concatenated and dimensionally reduced via UMAP. UMAP plots are shown, and Ki67 expression is overlayed. C) X-shift analysis was used to identify the expression level of each marker used in the clustering

bioRxiv preprint doi: https://doi.org/10.1101/2025.01.15.633135; this version posted January 19, 2025. The copyright holder for this preprint (which was not certified by peer review) is the author/funder, who has granted bioRxiv a license to display the preprint in perpetuity. It is made available under aCC-BY 4.0 International license.

performed in (B). D) Numbers of cells in each cluster from (B) were measured. E) Contribution of each genotype to each cluster was quantified. Statistical significance was tested by one-way ANOVA with Sidak's correction. *, ***, and **** correspond to p-values $\leq$ 0.05, 0.001, and 0.0001, respectively. N=3-4 mice/group and data shown are from 1 of 3 experiments.

Supplemental Figure 9. Testing HSPC survival and fitness. A) LTHSCs from WT and IL-27R$^{-/-}$ BM at 5 dpi was stained for Annexin V (AV) expression and free-amine staining viability dye (LD). This allowed the measurement of apoptotic (AV^{+}LD^{-}), late apoptotic (AV^{+}LD^{+}), and necrotic (AV^{-}LD^{+}) cells. The proportion of LTHSCs in each of these stages was quantified. B) Monocyte genotype was analyzed at 9 weeks post-transplant (wpt) from either naïve WT (CD45.1) or IL-27R$^{-/-}$ (CD45.1.2) donors (top) or from infected donors (bottom), including a 1:1 mix of infected WT and IL-27R$^{-/-}$ donors. Statistical significance was tested by Welch's t-test.

bioRxiv preprint doi: https://doi.org/10.1101/2025.01.15.633135; this version posted January 19, 2025. The copyright holder for this preprint (which was not certified by peer review) is the author/funder, who has granted bioRxiv a license to display the preprint in perpetuity. It is made available under aCC-BY 4.0 International license.

Supplemental Figure 1

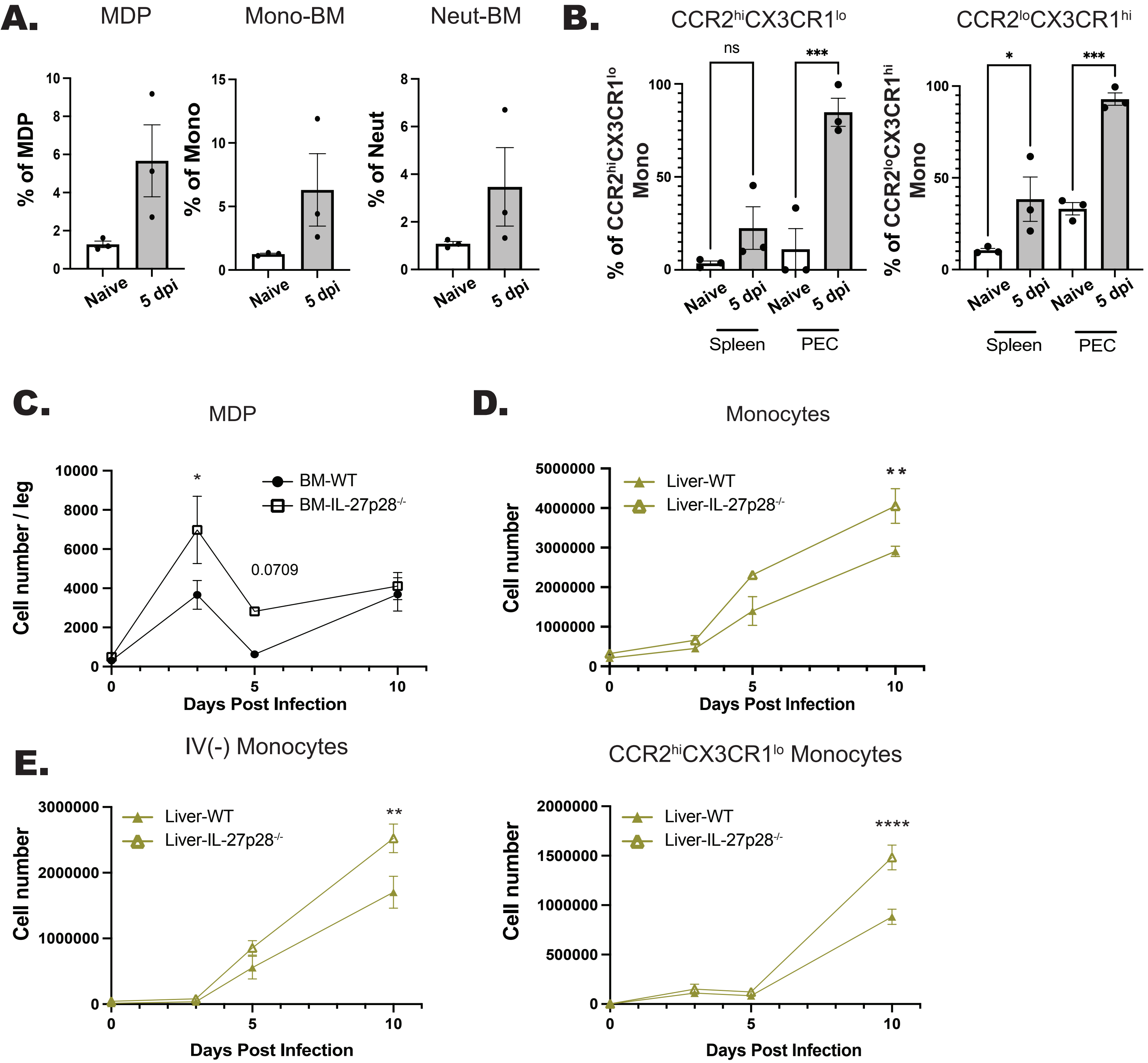

bioRxiv preprint doi: https://doi.org/10.1101/2025.01.15.633135; this version posted January 19, 2025. The copyright holder for this preprint (which was not certified by peer review) is the author/funder, who has granted bioRxiv a license to display the preprint in perpetuity. It is made available under aCC-BY 4.0 International license.

Supplemental Figure 2

Bone marrow gating strategy

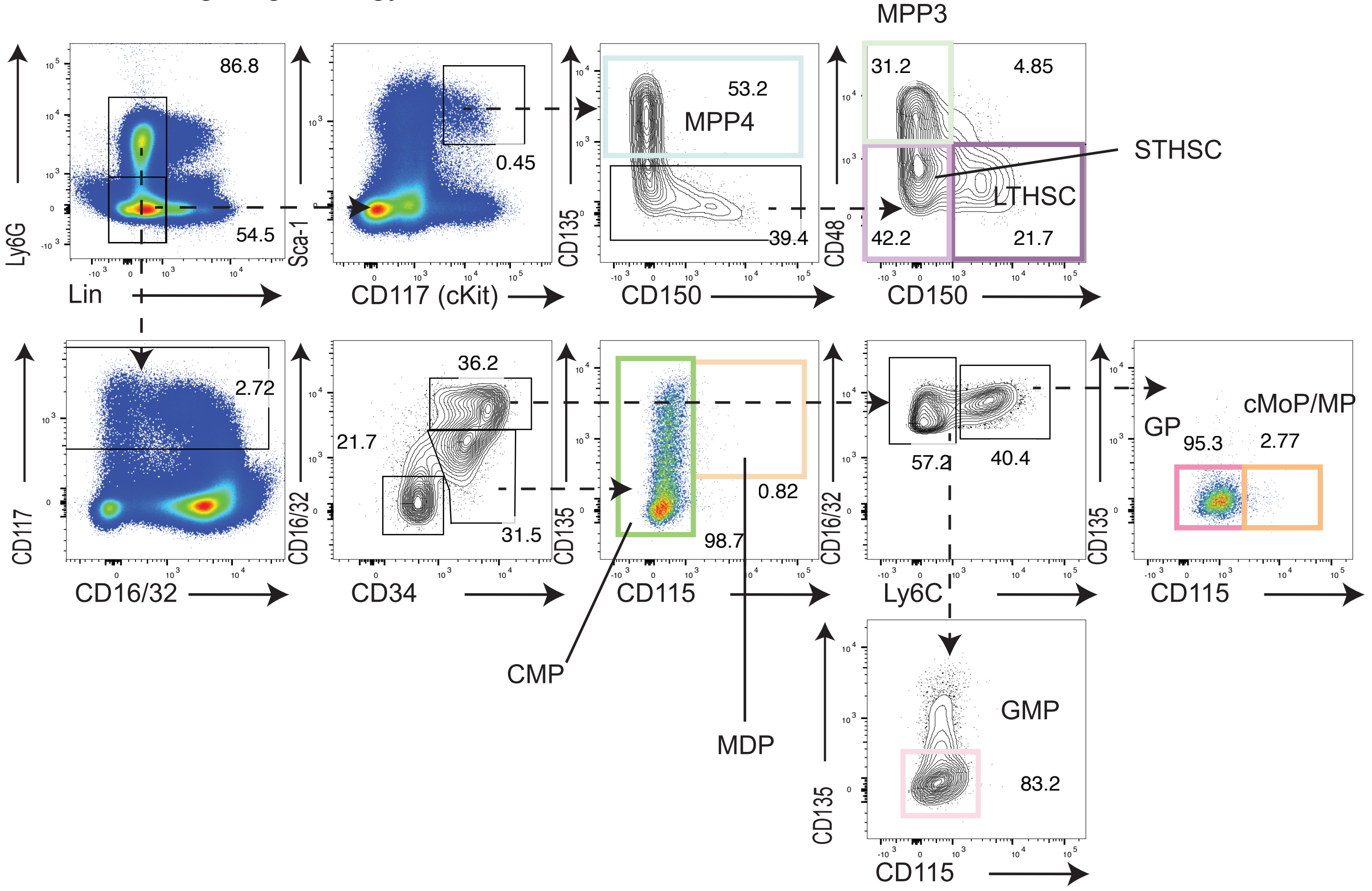

Periphery gating strategy

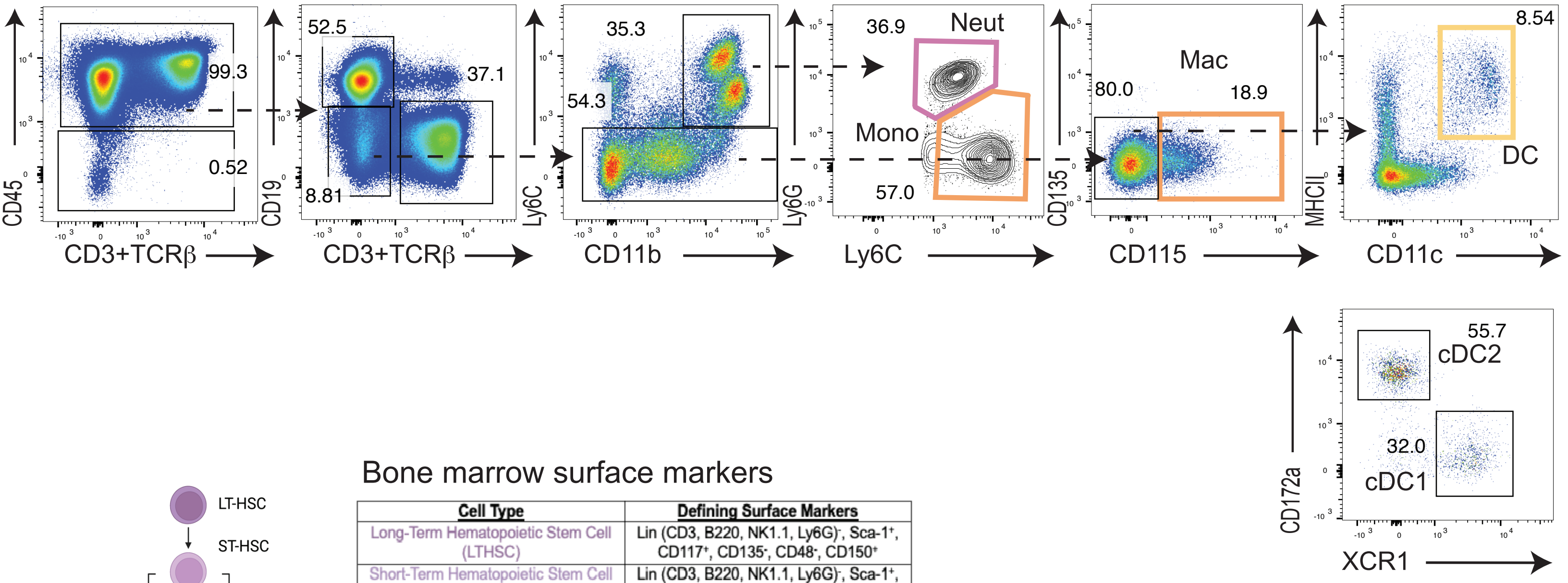

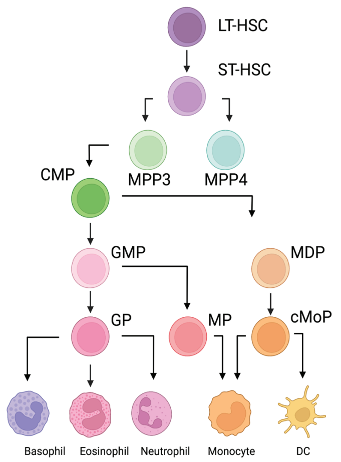

Bone marrow surface markers

Cell Type	Defining Surface Markers
Long-Term Hematopoietic Stem Cell (LTHSC)	Lin (CD3, B220, NK1.1, Ly6G)$^-$, Sca-1$^+$, CD117$^+$, CD135$^-$, CD48$^-$, CD150$^+$
Short-Term Hematopoietic Stem Cell (STHSC)	Lin (CD3, B220, NK1.1, Ly6G)$^-$, Sca-1$^+$, CD117$^+$, CD135$^-$, CD48$^-$, CD150$^-$
Multi-Potent Progenitor 3 (MPP3)	Lin (CD3, B220, NK1.1, Ly6G)$^-$, Sca-1$^+$, CD117$^+$, CD135$^-$, CD48$^+$, CD150$^-$
Multi-Potent Progenitor 4 (MPP4)	Lin (CD3, B220, NK1.1, Ly6G)$^-$, Sca-1$^+$, CD117$^+$, CD135$^+$
Common Myeloid Progenitor (CMP)	(CD3, B220, NK1.1)$^-$, CD117$^+$, CD34$^+$, CD16/32^{lo}, CD115$^-$
Monocyte-Dendritic cell Progenitor (MDP)	(CD3, B220, NK1.1)$^-$, CD117$^+$, CD34$^+$, CD16/32^{lo}, CD115$^+$
Common Monocyte Progenitor (cMoP)/ Monocyte Progenitor (MP)	(CD3, B220, NK1.1)$^-$, CD117$^+$, CD34$^+$, CD16/32^{hi}, Ly6C$^+$, CD135$^-$, CD115$^+$
Granulocyte-Monocyte Progenitor (GMP)	(CD3, B220, NK1.1)$^-$, CD117$^+$, CD34$^+$, CD16/32^{hi}, Ly6C$^-$, CD135$^-$, CD115$^-$
Granulocyte Progenitor (GP)	(CD3, B220, NK1.1)$^-$, CD117$^+$, CD34$^+$, CD16/32^{hi}, Ly6C$^+$, CD135$^-$, CD115$^-$

Periphery surface markers

Cell Type	Defining Surface Markers
T cells	CD45$^+$, CD19$^-$, CD3$^+$, TCRβ$^+$
B cells	CD45$^+$, CD19$^+$, CD3$^-$, TCRβ$^-$
Neutrophils	CD45$^+$, CD19$^-$, CD3$^-$, TCRβ$^-$, CD11b$^+$, Ly6C$^+$, Ly6G$^+$
Monocytes	CD45$^+$, CD19$^-$, CD3$^-$, TCRβ$^-$, CD11b$^+$, Ly6C$^+$, Ly6G$^-$
Macrophages	CD45$^+$, CD19$^-$, CD3$^-$, TCRβ$^-$, Ly6C$^-$, Ly6G$^-$, CD64$^+$
Dendritic Cells	CD45$^+$, CD19$^-$, CD3$^-$, TCRβ$^-$, Ly6C$^-$, Ly6G$^-$, CD64$^-$, CD11c$^+$, MHCII$^+$
Classical Dendritic Cells 1	CD45$^+$, CD19$^-$, CD3$^-$, TCRβ$^-$, Ly6C$^-$, Ly6G$^-$, CD64$^-$, CD11c$^+$, MHCII$^+$, XCR1$^+$, CD172a$^-$
Classical Dendritic Cells 2	CD45$^+$, CD19$^-$, CD3$^-$, TCRβ$^-$, Ly6C$^-$, Ly6G$^-$, CD64$^-$, CD11c$^+$, MHCII$^+$, XCR1$^+$, CD172a$^-$

bioRxiv preprint doi: https://doi.org/10.1101/2025.01.15.633135; this version posted January 19, 2025. The copyright holder for this preprint (which was not certified by peer review) is the author/funder, who has granted bioRxiv a license to display the preprint in perpetuity. It is made available under aCC-BY 4.0 International license.

Supplemental Figure 3

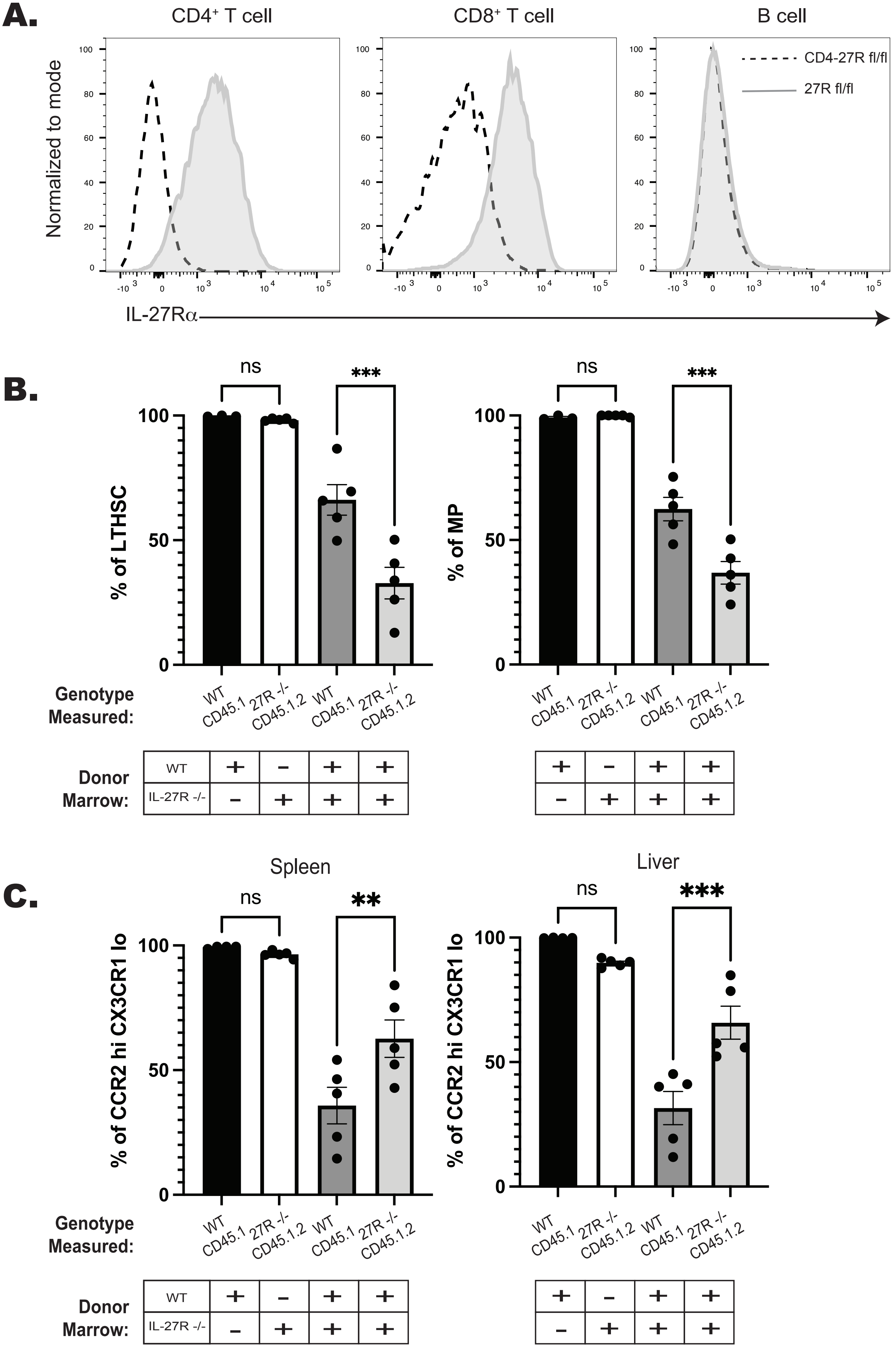

bioRxiv preprint doi: https://doi.org/10.1101/2025.01.15.633135; this version posted January 19, 2025. The copyright holder for this preprint (which was not certified by peer review) is the author/funder, who has granted bioRxiv a license to display the preprint in perpetuity. It is made available under aCC-BY 4.0 International license.

Supplemental Figure 4

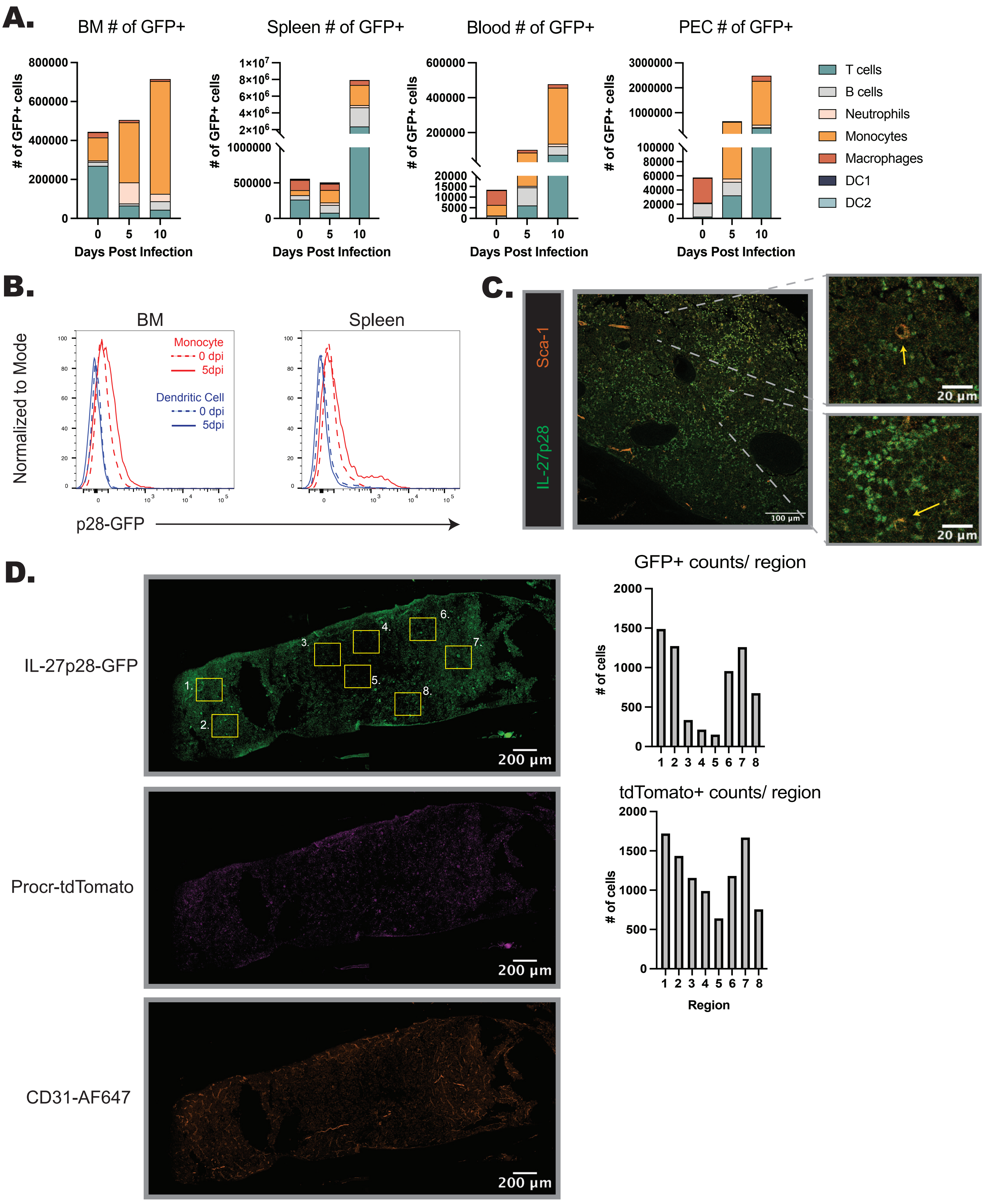

bioRxiv preprint doi: https://doi.org/10.1101/2025.01.15.633135; this version posted January 19, 2025. The copyright holder for this preprint (which was not certified by peer review) is the author/funder, who has granted bioRxiv a license to display the preprint in perpetuity. It is made available under aCC-BY 4.0 International license.

Supplemental Figure 5

A.

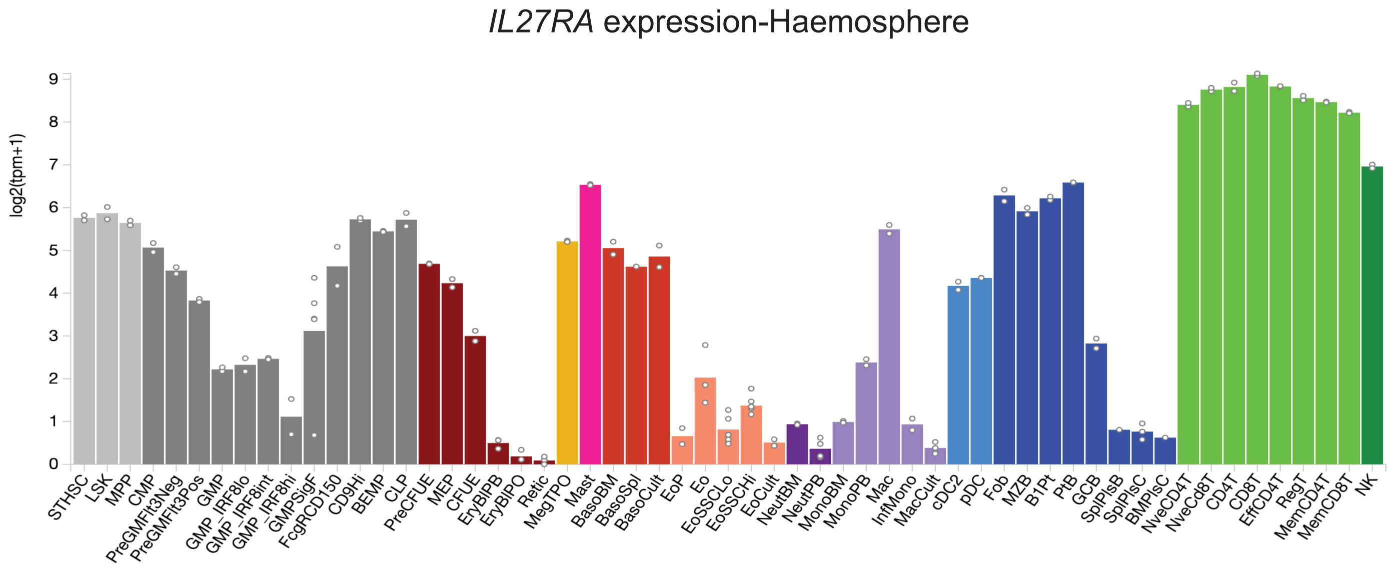

B.

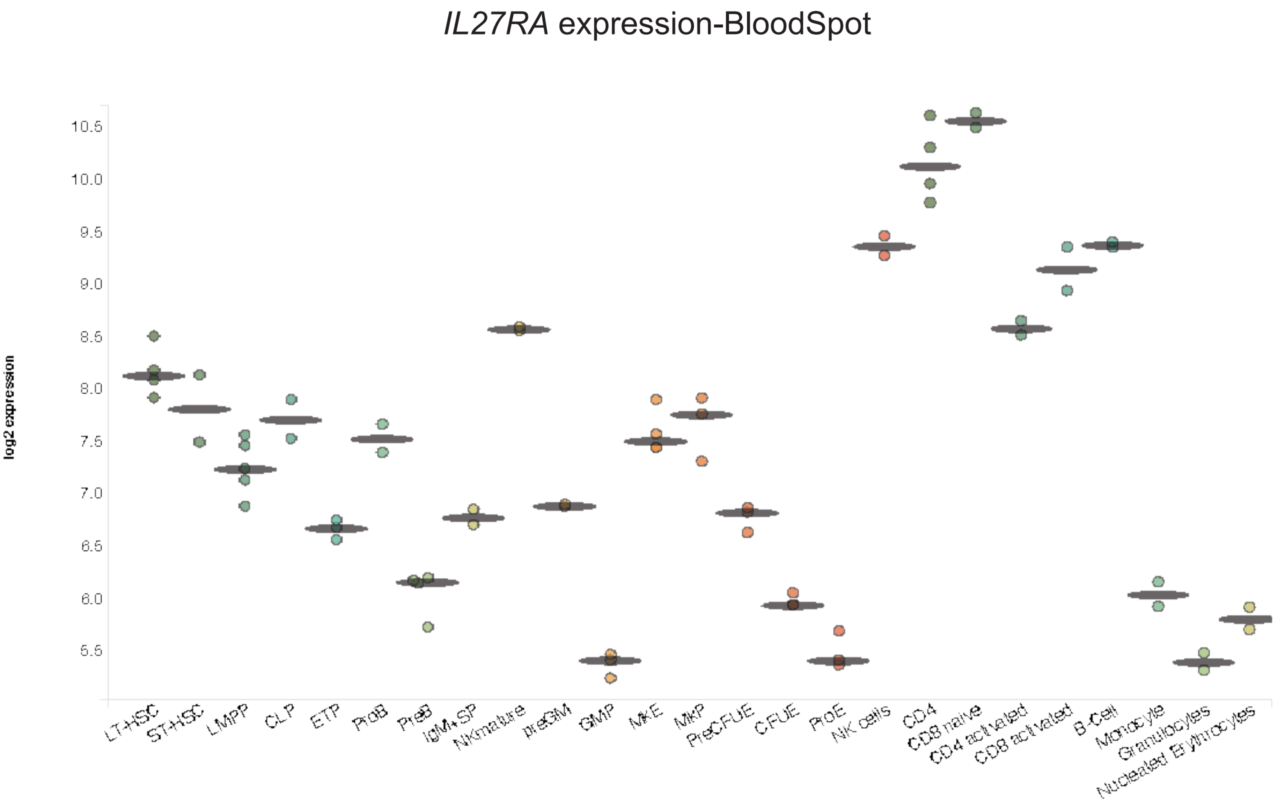

C.

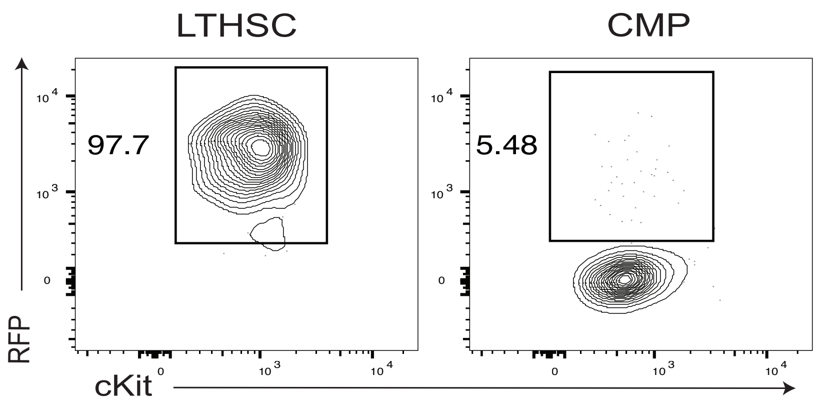

bioRxiv preprint doi: https://doi.org/10.1101/2025.01.15.633135; this version posted January 19, 2025. The copyright holder for this preprint (which was not certified by peer review) is the author/funder, who has granted bioRxiv a license to display the preprint in perpetuity. It is made available under aCC-BY 4.0 International license.

Supplemental Figure 6

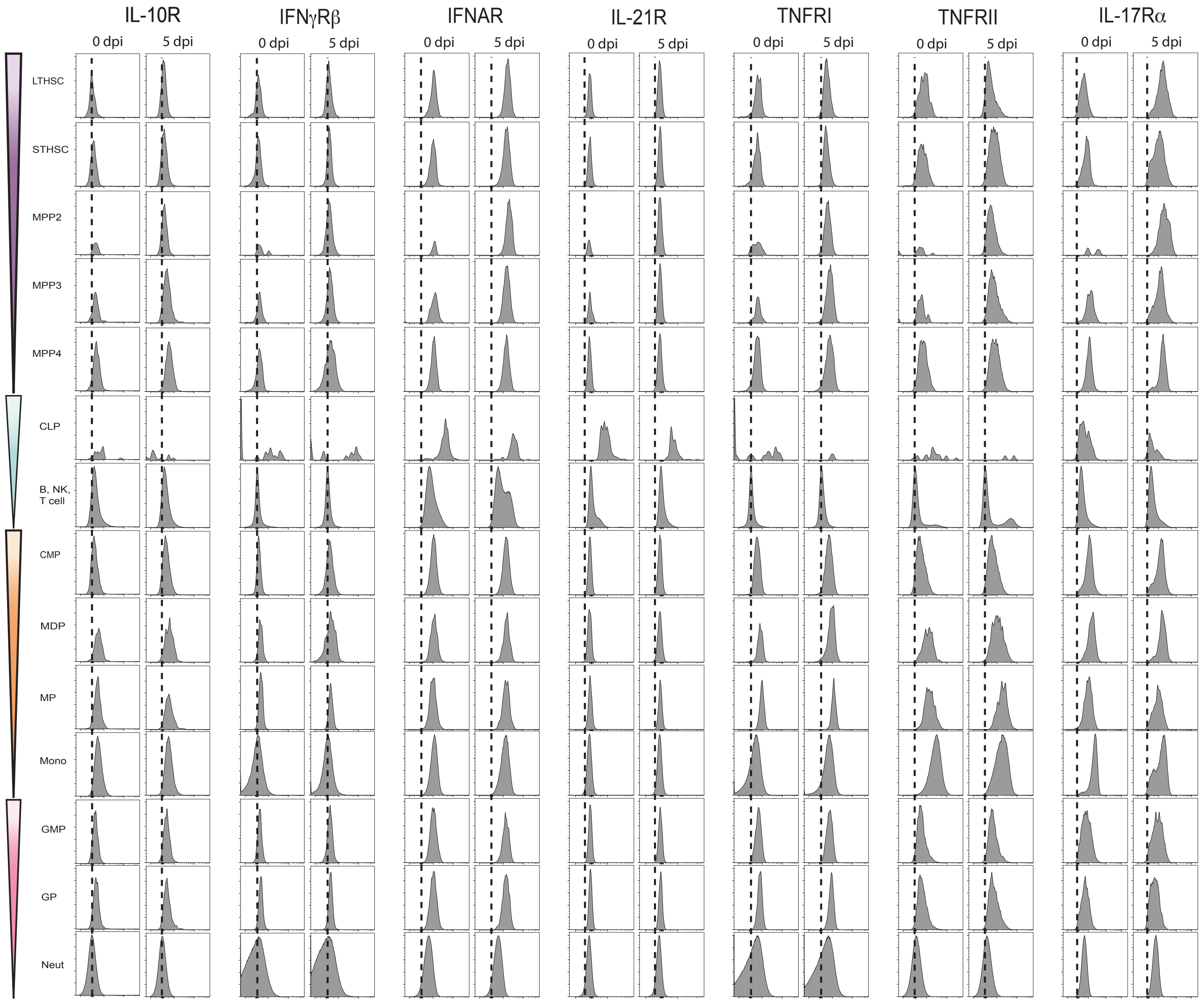

bioRxiv preprint doi: https://doi.org/10.1101/2025.01.15.633135; this version posted January 19, 2025. The copyright holder for this preprint (which was not certified by peer review) is the author/funder, who has granted bioRxiv a license to display the preprint in perpetuity. It is made available under aCC-BY 4.0 International license.

Supplemental Figure 7

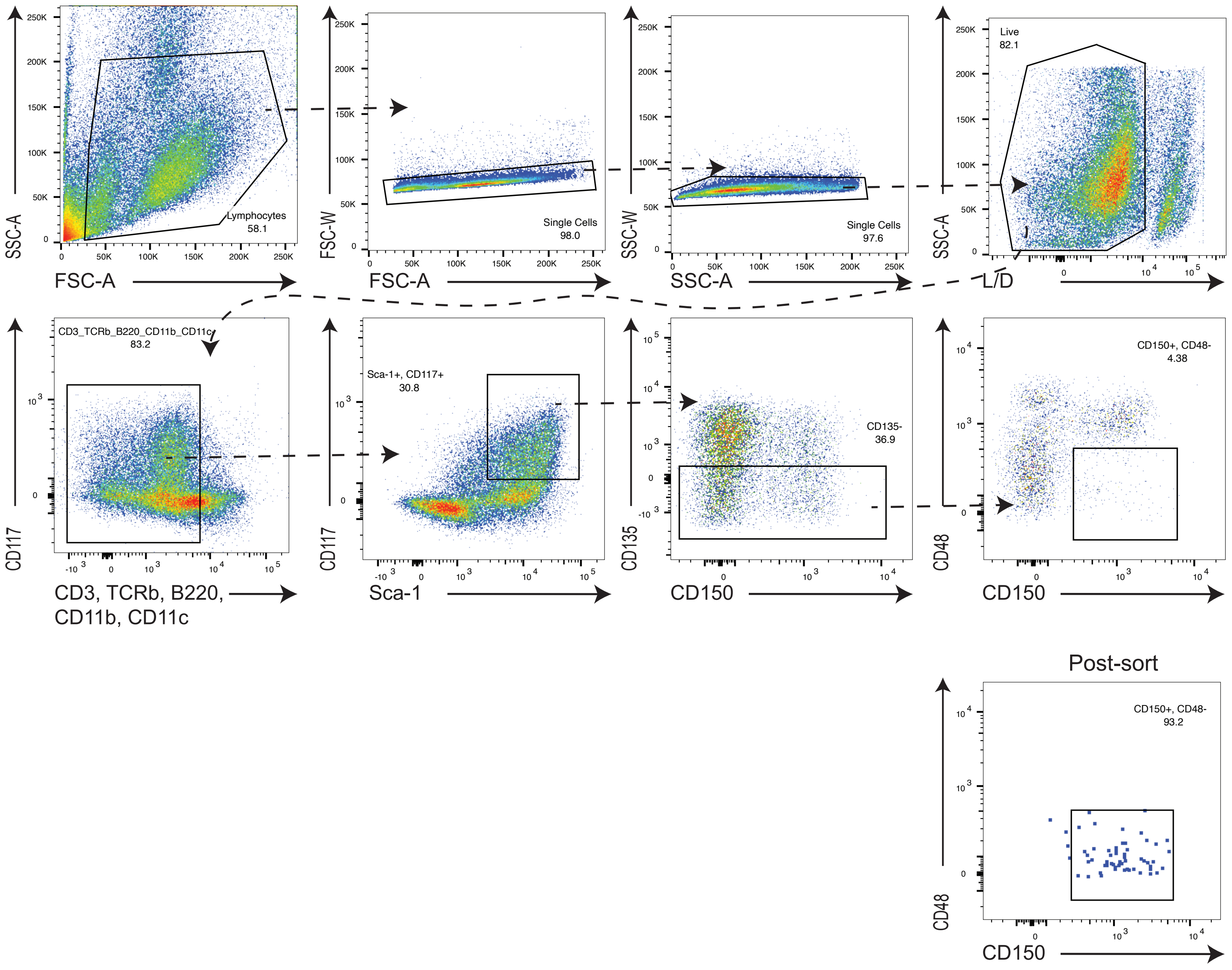

bioRxiv preprint doi: https://doi.org/10.1101/2025.01.15.633135; this version posted January 19, 2025. The copyright holder for this preprint (which was not certified by peer review) is the author/funder, who has granted bioRxiv a license to display the preprint in perpetuity. It is made available under aCC-BY 4.0 International license.

Supplemental Figure 8

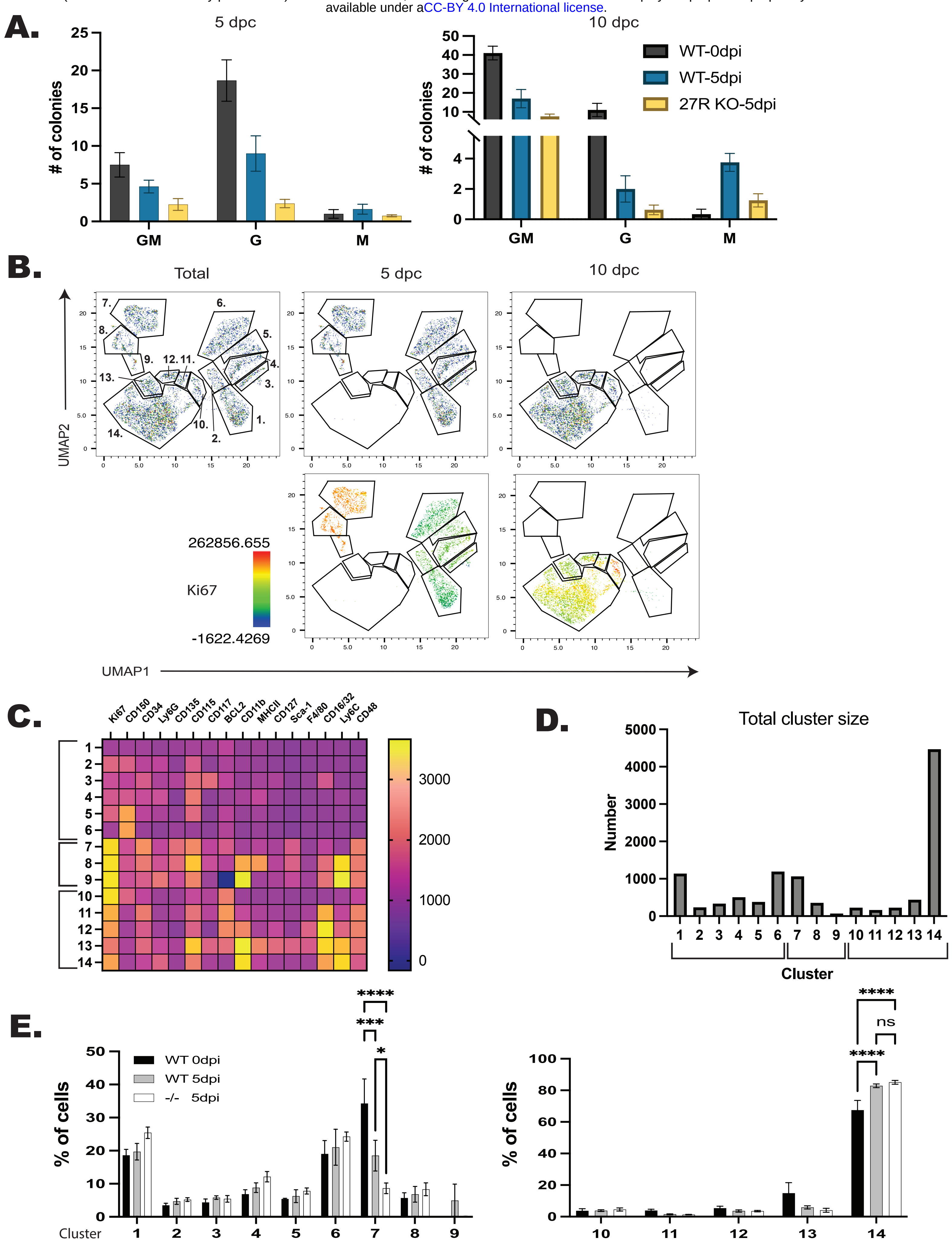

bioRxiv preprint doi: https://doi.org/10.1101/2025.01.15.633135; this version posted January 19, 2025. The copyright holder for this preprint (which was not certified by peer review) is the author/funder, who has granted bioRxiv a license to display the preprint in perpetuity. It is made available under aCC-BY 4.0 International license.

Supplemental Figure 9

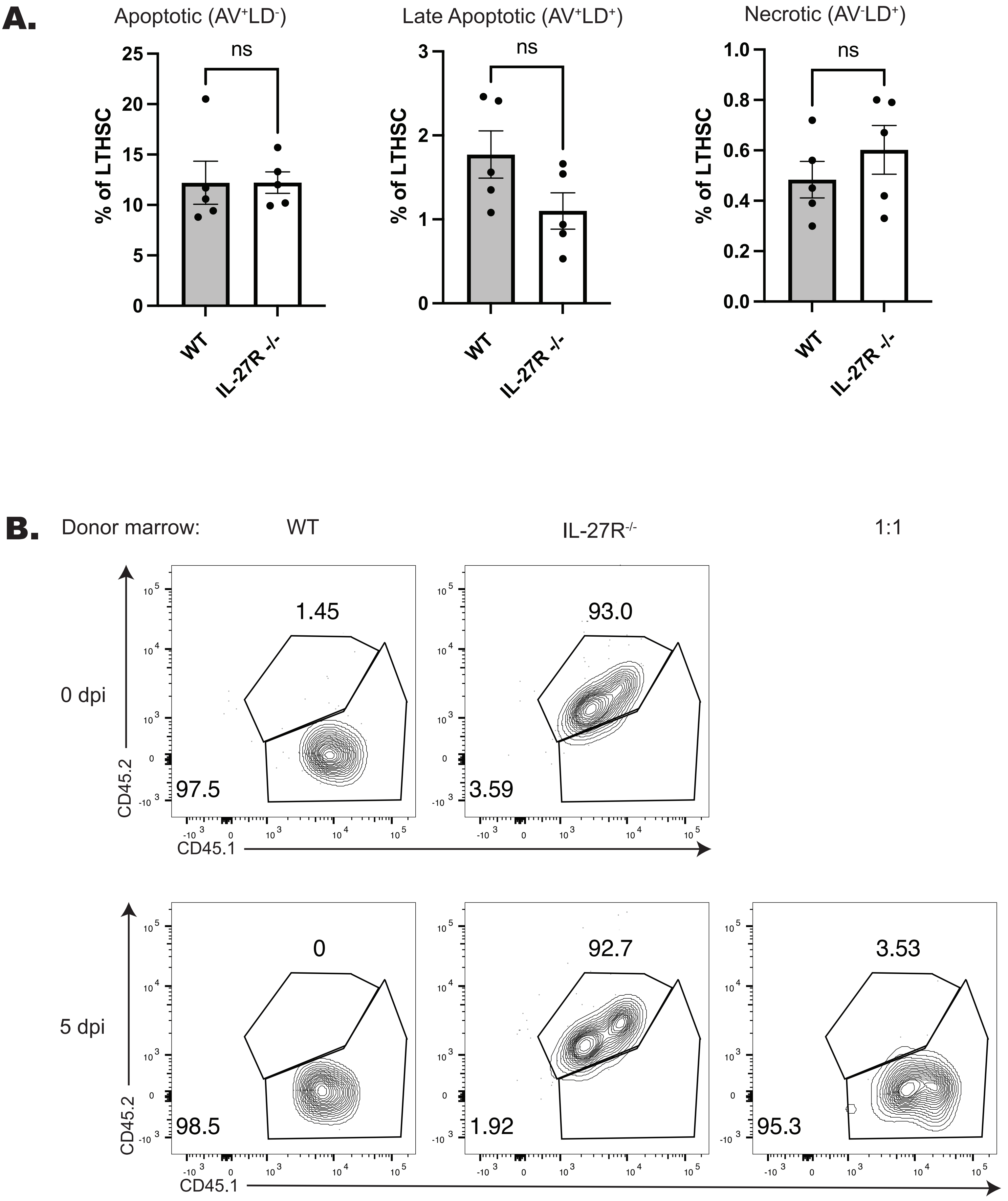

Supplement: Supplementary file 1 [file NIHPP2025.01.15.633135v1-supplement-1.pdf]
